# Immaturity of Visual Fixations in Dyslexic Children

**DOI:** 10.3389/fnhum.2016.00058

**Published:** 2016-02-17

**Authors:** Aimé Tiadi, Christophe-Loïc Gérard, Hugo Peyre, Emmanuel Bui-Quoc, Maria Pia Bucci

**Affiliations:** ^1^UMR 1141, Institut National de la Santé et de la Recherche Médicale - Université Paris Diderot - Paris 7, Robert Debré HospitalParis, France; ^2^Child and Adolescent Psychiatry Department, Robert Debré HospitalParis, France; ^3^Ophthalmology Department, Robert Debré HospitalParis, France

**Keywords:** visual fixation, frontal eye field (FEF), superior colliculus (SC), immaturity, dyslexia, children, eye movements, attention

## Abstract

To our knowledge, behavioral studies recording visual fixations abilities in dyslexic children are scarce. The object of this article is to explore further the visual fixation ability in dyslexics compared to chronological age-matched and reading age-matched non-dyslexic children. Fifty-five dyslexic children from 7 to 14 years old, 55 chronological age-matched non-dyslexic children and 55 reading age-matched non-dyslexic children participated to this study. Eye movements from both eyes were recorded horizontally and vertically by a video-oculography system (EyeBrain^®^ T2). The fixation task consisted in fixating a white-filled circle appearing in the center of the screen for 30 s. Results showed that dyslexic children produced a significantly higher number of unwanted saccades than both groups of non-dyslexic children. Moreover, the number of unwanted saccades significantly decreased with age in both groups of non-dyslexic children, but not in dyslexics. Furthermore, dyslexics made more saccades during the last 15 s of fixation period with respect to both groups of non-dyslexic children. Such poor visual fixation capability in dyslexic children could be due to impaired attention abilities, as well as to an immaturity of the cortical areas controlling the fixation system.

## Introduction

Visual fixation consists in maintaining an image on the fovea in order to perceive it. Palvidis (1981) was the first to report poor fixation capabilities in 12 dyslexic children with respect to 12 non dyslexic children. More recently, De Luca et al. (1999) explored fixation capability in 10 dyslexic children and in 11 non-dyslexic children of comparable chronological age and they did not find any difference between the two groups of children. These authors suggested that oculomotor differences between dyslexic and non-dyslexic children occurred during reading task only. In the present study, we explored further fixation capability in a large number of dyslexic children and we compared their results with those of two groups of non-dyslexic children having similar chronological and reading age.

Munoz and Wurtz (1992) showed in monkeys that neural cells located in the rostral cells are activated to avoid unwanted saccades. These authors recorded fixation activity and saccade suppression in two monkeys 48 h after muscimol injection in the rostral cells of the superior colliculus (SC), and showed that muscimol inhibits the neural transmission of the rostral cells. Indeed, after injection of muscimol, the monkeys had more difficulty suppressing saccade initiation and several unwanted saccades were initiated before the target switched on. This study showed that rostral cells of the SC are involved in both visual fixation and saccade suppression. In 1995, the same authors explored further the role of SC cells in monkeys and they confirmed that some cells in the rostral pole are responsible for visual fixation while the other cells are responsible for preparation and generation of saccades (Munoz and Wurtz, 1995).

The frontal eye field (FEF) is known to play also an important role in the control of saccades and in their suppression (Leigh and Zee, 2006). For instance, Burman and Bruce (1997) studied suppression of saccades using an electrical micro-stimulation in monkeys eliciting several types of saccades (visual-guided, memory, pro- and anti-saccades). These authors reported that the ventral-lateral area of the FEF, near the arcuate spur, contains a specific and delimited region responsible for saccade suppression. Furthermore, they confirmed previous findings from Segraves and Park (1993) suggesting that the control and inhibition of the saccades is done by the relationship between FEF cells, SC and pontine-omnipause region.

Hasegawa et al. (2004) recorded the activity of the neurons in the prefrontal cortex of monkeys in order to explore how this area is involved in the suppression of unwanted saccades. They used an oculomotor task in which monkeys had to remember a stimulus location, i.e., to avoid making a saccade, and they observed a group of neurons in both the FEF and the caudal prefrontal cortex that were activated during saccade suppression.

In patients with frontal lesions, Guitton et al. (1985) reported more unwanted saccades than among control subjects, suggesting that frontal lobes contribute to the suppression of inappropriate saccades. Moreover, Braun et al. (1992) recorded saccades in adult patients with frontal and parietal lesions vs. control subjects, and they showed that patients had greater difficulties suppressing saccades, suggesting a deficit in the fixation control system.

Behavioral studies recording fixation capabilities in children are, to our knowledge, scarce. Biscaldi et al. (1996) compared oculomotor capability in dyslexic and non-dyslexic children and adults (from 12 to 32 years old) and they showed that dyslexics had more express saccades than non-dyslexic subjects, suggesting a poor fixation control among dyslexic subjects. These authors advanced the hypothesis that the deficit in attentional process might be linked with the parietal cortical areas in the dyslexic population. Recently, our group explored the quality of fixation during a dual postural/oculomotor task in both healthy (Ajrezo et al., 2013) and dyslexic children (Bucci et al., 2014). Ajrezo et al. (2013) reported that the quality of fixation during a dual task improved with the increasing age of the children; the number of saccades during fixation was significantly reduced in adolescents, suggesting that the fixation performance in children is underdeveloped, due to the lack of maturation of their cortical areas (Luna and Sweeney, 2001). In line with this thinking, Bucci et al. (2014) studied the quality of fixation during a dual task in 30 dyslexics (from 7 to 13 years old) compared with 30 chronological age-matched and 30 reading age-matched non-dyslexic children. The authors showed poor quality of the fixation in dyslexic children compared to both non-dyslexic groups, which could be in relation with visual attention deficits reported in dyslexic children (Facoetti et al., 2003).

Based on all these findings, the goal of the present study was to further explore visual fixation capability in dyslexic children and to compare these results to those from two groups of chronological and reading age-matched non-dyslexic children. This was done in order to explore further whether fixation instability in dyslexic children was due to the consequence of a reduced reading exposure or rather due to immaturity of cortical areas involved in the visual fixation control.

## Materials and Methods

### Subjects

Fifty-five dyslexic children from 7 to 14 years old (mean age: 10.1 ± 0.2 years) participated to the study. Dyslexic children were recruited from Robert Debré Pediatric Hospital, to which they had been referred for a complete evaluation of their dyslexia including an extensive examination of their neurological/psychological and phonological capabilities. For each child, we measured the time they required to read a text passage, assessed their general text comprehension, and evaluated their ability to read words and pseudo-words using the L2MA battery (Chevrie-Muller et al., 1997). This is the standard test in France. It was developed by the “Centre de Psychologie Appliquée de Paris” and has been used previously by our team (Bucci et al., 2012, 2013a,b, 2014; Tiadi et al., 2014) to select dyslexic populations. Inclusion criteria were scored on the L2MA which were more than two standard deviations from the mean, and a normal mean intelligence quotient (IQ, evaluated using the WISC-IV), namely between 85 and 115. Any hyperactivity deficit has been excluded by using the ADHD Rating Scale-parental report (ADHD-RS).

Fifty-five chronological and reading age-matched non-dyslexic children respectively of 10.4 ± 0.3 years old and 8.14 ± 0.20 years were also examined. The inclusion criteria were as follows: no known neurological or psychiatric abnormalities, no history of reading difficulty, no visual impairment, or difficulty with near vision. Also, IQ in controls was estimated on two subtests, one assessing the verbal capability (similarities test) and one assessing the logic capability (matrix reasoning test). Normal range for both tests is 10 ± 3 (Wechsler intelligence scale for children—fourth edition, 2004). All the healthy children we tested had normal verbal (10.36 ± 0.8) and logic capabilities (10.64 ± 0.5). The reading age-matched of all children were assessed by using the ELFE test (cogni-sciences, Grenoble) and Table 1 shows the reading age for each dyslexic children tested.

**Table 1:** **Chronological and reading age of each dyslexic child tested**.

Dyslexics	Chronological age SE ± 0.2	Reading age SE ± 0.2	Dyslexic	Chronological age SE ± 0.2	Reading age SE ± 0.2
D1	7.42	6	D29	9.30	7.5
D2	7.60	6	D30	9.70	7.5
D3	7.70	6	D31	10.25	8
D4	7.70	6	D32	10.83	8
D5	7.75	6	D33	10.10	8
D6	7.80	6	D34	10.80	8
D7	7.92	6	D35	10.30	8
D8	8.00	6	D36	10.50	8
D9	8.10	6	D37	11.42	9
D10	8.20	6	D38	11.58	9
D11	8.20	6	D39	11.42	9
D12	8.25	7	D40	11.50	9
D13	8.30	7	D41	11.50	9
D14	8.33	7	D42	11.17	9
D15	8.33	7	D43	11.10	9
D16	8.40	7	D44	11.20	9
D17	8.50	7	D45	12.08	10
D18	8.60	7	D46	12.17	10
D19	8.67	7	D47	12.10	10
D20	9.17	7	D48	13.42	11
D21	9.17	7	D49	13.00	11
D22	9.40	7	D50	13.75	11
D23	9.40	7	D51	13.58	11
D24	9.42	7	D52	13.00	11
D25	9.42	7	D53	13.90	11
D26	9.75	7	D54	14.33	12
D27	9.83	8	D55	14.67	12
D28	9.90	7

The investigation adhered to the principles of the Declaration of Helsinki and was approved by our Institutional Human Experimentation Committee. Written consent was obtained from the children’s parents after they were given an explanation about the experimental procedure.

### Ophthalmologic and Orthoptic Evaluation

All children had normal values for ophthalmologic and orthoptic examination (showed in Table 2). The corrected visual acuity was normal (≥20/20) for all children. All children had normal binocular vision evaluated with the TNO random dot test. The near point of convergence (NPC) was normal for all children. Heterophoria (i.e., the latent deviation of one covered eye when the other is not covered) measured using the cover–uncover test at near distance (30 cm) was normal for all children. Fusional amplitudes of convergence and divergence were measured at near distance (30 cm) using a base-in and a base-out prism bar; dyslexic children showed poorer convergence and divergence capabilities than the two groups of non-dyslexic children.

**Table 2:** **Clinical characteristics of children tested**.

Children (years)	TNO (s of arc)	PPC (cm)	Phoria (pD)	Convergence (pD)	Divergence (pD)
Dyslexics (10.2 ± 0.2)	60 ± 10.4	3.00 ± 2.2	−2.2 ± 0.7	26.70 ± 1.0	10.60 ± 0.5
Reading age-matched non-dyslexics (8.34 ± 0.20)	60 ± 9.5	2.00 ± 2.0	−2.4 ± 0.5	34.00 ± 1.2	16.02 ± 1.2
Chronological age-matched non-dyslexics (10.5 ± 0.3)	66 ± 15.5	2.18 ± 2.9	−2.5 ± 1.5	35.00 ± 0.8	14.60 ± 0.3

*Note: clinical characteristics of the dyslexic and non-dyslexic children examined. Mean values ± standard error of: binocular vision (Stereoacuity test, TNO measured in seconds of arc); near point of convergence (NPC measured in cm); Heterophoria at near distance measured in prism diopters (negative values indicate exophoria); Vergence fusional amplitudes (convergence and divergence) at near distance measured in prism diopters*.

### Procedure

Stimulus was presented on a 22^″^ PC screen with a resolution of 1920 × 1080 and a refresh rate of 60 Hz. The child was seated in a chair in a dark room, with his/her head stabilized by a forehead and chin support. Viewing was binocular; the viewing distance was 60 cm. Calibration was done at the beginning of the fixation task. During the calibration procedure, the children were asked to fixate a grid of 13 points (diameter 0.5°) mapping the screen. Each calibration point required a fixation of 250 ms to be validated. A polynomial function with five parameters was used to fit the calibration data and to determine the visual angles. Afterwards, the child was invited to fixate a white-filled circle subtending a visual angle of 0.50° appearing in the center of the screen during 30 s. Two fixation tasks were recorded for each child.

### Eye Movement Recording

Eye movements were recorded binocularly; horizontal and vertical eye positions were recorded independently and simultaneously for each eye with the EyeBrain T2, an eye-tracking device CE-approved for medical applications. Recording frequency for both eyes was set up to 300 Hz.

### Data Processing

Calibration factors for each eye were determined from the eye positions during the calibration procedure (see Bucci et al., 2012). MeyeAnalysis^®^ software (provided with the eye tracker) was used to extract the number of saccades during the fixation task. This software automatically detects both the onset and the offset of each saccade by using a built-in saccade detection algorithm. All detected saccades were manually checked by the investigator and corrected/discarded if necessary. All saccades ≥2° were counted given that it is well known that micro-saccades are normally smaller than such amplitude (Krekelberg, 2011). For each child (dyslexic and non-dyslexic) we counted the number of saccades measured in the two fixation tasks. Also, in order to evaluate visual attention in children, the number of saccade was also recorded in the first and in the last 15 s of fixation recording.

### Statistical Analysis

Data were analyzed using the linear regression models for the three groups of children separately (dyslexic and chronological and reading age-matched non-dyslexic); dependant variable was the number of saccades measured during fixation task and predictor variable was the children age (in years). An analysis of variance (ANOVA) was also performed with groups as inter-subject factor and the number of saccades measured during fixation task (during all 30 s and also during the first and in the last 15 s of recording) as within-subject factors. *Post hoc* comparisons were made with the Fischer’s test (LSD). The effect of a factor is significant when the *p*-value is below 0.05.

## Results

**Figure 1:**
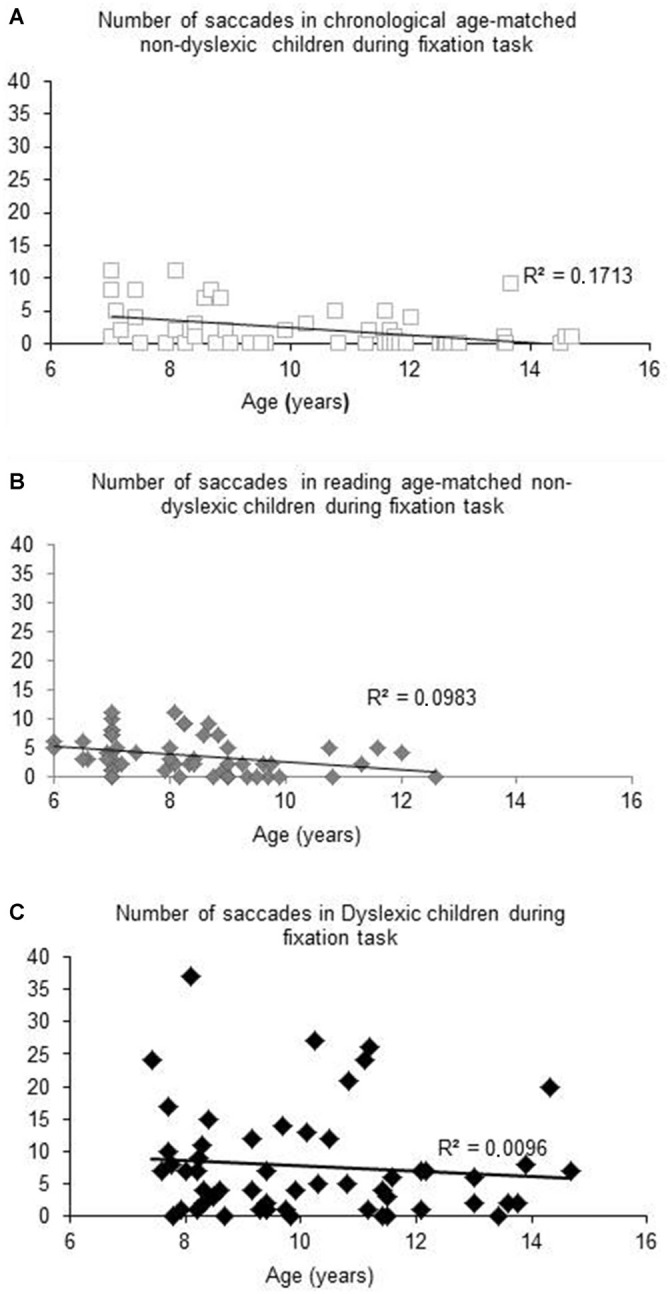
**Number of saccades measured during fixation task for Chronological age-matched non-dyslexic children (A), reading age-matched non-dyslexic children (B) and dyslexic children (C).** Line represents the corresponding regression.

Figure 1 shows the number of saccades measured during the total fixation task period as a function of the age (in years) of each child tested and the regression line for both chronological (A) and reading age-matched non-dyslexic children (B) and dyslexic children (C). For both groups of non-dyslexic children, the number of saccades decreased significantly with the age of the children (*R*^2^ = 0.1727, *p* < 0.02 and *R*^2^ = 0.0983, *p* < 0.0001, respectively for chronological and reading age-matched non-dyslexic children). In contrast, for dyslexic children, the number of saccades did not decrease significantly with their chronological age (*R*^2^ = 0.0096, *p* = 0.47) neither with their reading age (*R*^2^ = 0.031, *p* = 0.19).

**Figure 2:**
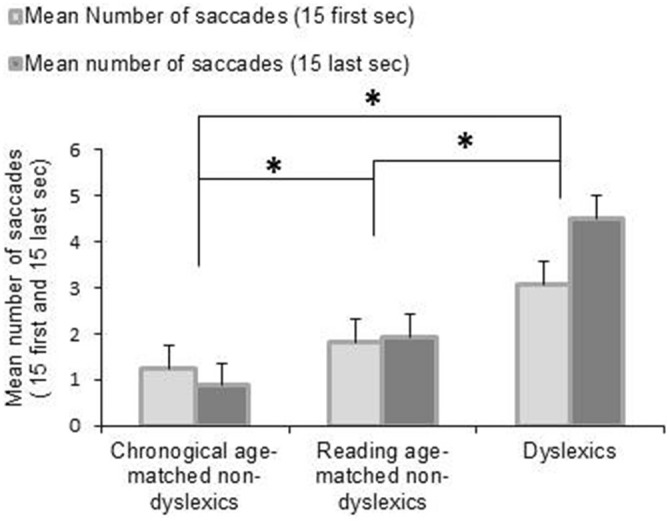
**Mean values of number of saccades during fixation task for the first 15 and the last 15 s.** Vertical bars indicate the standard error. Asterisk indicates a significant difference between the three groups of children.

Figure 2 shows the mean number of saccades during the first 15 and the last 15 s of the fixation period for each group of children tested. The ANOVA revealed a main effect of group reflecting that the dyslexic readers generally made more saccades than the control groups (*F*_(2,162)_ = 14.999, *p* < 0.0001). Moreover, we found an interaction between groups of children and period of fixation showing that dyslexic children made more saccades during the final half of the task than during the initial half (*p* < 0.0001)—in the other two groups this was not the case. *Post hoc* test indicated also that dyslexic group made significant more number of saccades during the first 15 s of fixation with respect to the other two groups of non-dyslexic children (all *p* < 0.0001).

In order to know further the proportion of dyslexic children who made frequent saccades during fixation we showed in Table 3 the range and the different percentage of the number of saccades made by each group of children. Dyslexic children made the largest range of saccades (0–29 saccades) compared to the other two groups of children (0–11 saccades for chronological age-matched non-dyslexics group and 0–13 saccades for reading age-matched non-dyslexics group). Furthermore, the majority of the chronological age-matched control children (78%) and 58% of reading age-matched children made three or less saccade in the fixation task. In contrast, about the half of dyslexic children (42%) made three or less saccade in the fixation task while 58% of them made more than three saccades.

**Table 3:** **Percentage of the number of saccades for each group tested**.

Range of saccades	Chronological age-matched non-dyslexic children (%)	Reading age-matched non-dyslexic children (%)	Dyslexics
0	49	24	15
1	9	7	7
2	15	16	11
3	5	11	9
4	4	5	9
5	4	11	2
6	0	2	4
7	4	7	9
8	4	4	4
9	4	5	2
10	0	2	2
11	4	4	4
12	0	0	2
13	0	2	2
14	0	0	5
15	0	0	0
16	0	0	0
17	0	0	2
18	0	0	0
19	0	0	2
20	0	0	0
21	0	0	2
22	0	0	0
23	0	0	0
24	0	0	2
25	0	0	0
26	0	0	2
27	0	0	2
28	0	0	0
29	0	0	4
Total	100	100	100

*Note: Range and different percentages of the number of saccades produced by each group of children tested*.

## Discussion

The main findings of this study are as follows: (i) the number of saccades during fixation is significantly higher in dyslexic children with respect to non-dyslexic children groups and (ii) the number of saccades during fixation significantly decreases with age in non-dyslexic children groups only. These findings are discussed below.

### The Number of Saccades During Fixation is Significantly Higher in Dyslexic with Respect to Non-Dyslexic Children

Our data revealed that dyslexic children made a higher number of saccades during fixation than both non-dyslexic children groups. This study enlarges the previous work of Eden et al. (1994) in which poor fixation capability in 26 dyslexic children (11 years old) has been reported without counting the number of saccades during fixation task.

Our finding suggests that dyslexic children could have a weak ability to suppress unwanted saccades, most likely due to their difficulty in inhibition processing as well as to attentional deficits, even if we excluded in this study dyslexic with attentional deficiencies (see “Materials and Methods” Section). Such attentional impairment has been previously reported by Biscaldi et al. (1996), Facoetti et al. (2003) and de Araujo et al. (2015). More recently, Ruffino et al. (2010) studied attentional engagement in 28 dyslexics and 55 non-dyslexic children through a spatio-temporal attentional task with a fixation mark. Authors showed that dyslexic children only significantly exhibited an impaired identification of the targets and suggested that such impairment was linked to the attentional engagement deficit. Also, during single word reading, Thaler et al. (2009) reported that dyslexic children with attentional deficits made longer fixations than dyslexic without attentional deficits. In the present study, such attentional difficulty could be also substained by the fact that dyslexic children made significantly more saccades in the last 15 s during fixation task, while the two other groups made almost the same number of saccade in the first 15 and last 15 s.

We also suggested that structures of the cortex responsible for saccade suppression and fixation control are immature in dyslexic children. Indeed, our data showed that reading age-matched non-dyslexic children made smaller number of saccades than dyslexics suggesting that the reading experience does not explain the poor fixation reported in the present study in dyslexic children. These findings are in line with Clark et al.’s (2014) study concerning the neuroanatomical structures in dyslexics. These authors acquired reading and spelling data longitudinally via a RMI scans before the onset of reading learning and after dyslexia was diagnosed. They found that the visual area cortices were thinner in children who developed dyslexia. So, the cortical immaturity we suggested concerning the poor fixation in dyslexic children could be strictly linked with poor activity of SC and FEF neurons for a correct control of visual fixation. Several studies demonstrated the key role of SC and FEF in attention processing. In this line, Lovejoy and Krauzlis (2010) recorded the activity of SC in two monkeys after a muscimol injection and they reported a poor ability for them to focus their attention on a central fixation, suggesting that SC plays an important role in the selective attention. Krauzlis et al. (2013) also reported that SC is involved in spatial overt and covert attention. According to these authors, SC activity leads to spatial attention regulation during overt orienting eye movements. Basing their analysis on Ignashchenkova et al.’s (2004) study in which the shifts of attention in monkeys were analyzed, Krauzlis et al. (2013) also indicated the high activity of visual-motor SC neurons during covert shifts of attention. Thus, SC is important both in motor consequences as well as in visual-motor processing of attention. On the other hand, FEF neurons are known also to be involved in visual attention. Moore and Fallah (2001) simulated the FEF area in two monkeys while the latter performed a spatial attention task. The results showed that the enhancement of spatial attention is strongly related to the increase of FEF neurons activity and this has been confirmed in other studies on monkeys (Moore and Fallah, 2004) as well as on healthy subjects and patients with FEF lesions (Pierrot-Deseilligny et al., 2004; Esterman et al., 2015).

### The Number of Saccades During Fixation Significantly Decreases with Age in Non-Dyslexic Children Only

This study showed that the number of saccades in both chronological and reading age-matched non-dyslexic groups significantly decreased with age, suggesting that the quality of the fixation improves with age. This finding is in line with previous studies exploring fixations in children populations (Munoz et al., 1998; Ajrezo et al., 2013) and showing that the quality of visual fixation in younger children is poor and improves until adolescence. Thus, the frontal and prefrontal cortices involved in fixation abilities as well as in saccade suppressions are still developing in young children. Other neurophysiological and neurocognitive studies (Sharpe and Zackon, 1987; Barkovich, 2000; Luna et al., 2004, 2008) showed that brain maturation is reached during adolescence (14 years old). Consequently we could make the hypothesis that the improvement of the visual fixation capabilities with age is related to a gradual and progressive maturation of such cortical structures until adolescence. On the other hand, the improvement of the quality of visual fixation with age could also be correlated with the maturation of visual attention capabilities in children. Indeed, it is well known that the attentional functions are immature in children. Konrad et al. (2005) investigated the neural mechanisms of attention in children from 8 to 12 years old using fRMI to assess the neural attentional networks. These networks involved right fronto-parietal regions for alerting, right temporo-parietal junction and right inferior frontal gyrus for orienting and reorienting, as well as anterior cingulate and lateral prefrontal cortex for executive attention. The results showed a weak activity of the attention regions that were assessed but an important activity of the superior areas and the occipital cortex. The authors concluded that the attentional functions are not mature but are in transition in children. These results could explain the weak inhibition capabilities in children from 8 to 12 years old, as was reported previously by Bunge et al. (2002).

Based on all these findings, we suggested that the immaturity of these cortical areas in dyslexic children could lead to difficulties for them to focus their attention on the central target, leading to poor visual fixation performances compared with non-dyslexic age-matched children.

Finally, such poor fixation performance in dyslexic children could have a negative effect during reading given that during fixation period children extract lexical information from the words. So, such visual information processing could be responsible for longer fixations during reading, as reported by Bucci et al. (2012).

## Conclusion

Our findings showed that dyslexic children exhibited many difficulties fixating a visual target and therefore triggered significantly more unwanted saccades than age-matched non-dyslexic children. An immaturity of cortical areas is most likely responsible for such poor fixation capabilities in dyslexic children. Orthoptic as well as visuo-attentional training in dyslexic children could help them to better focus their attention and therefore decrease reading errors and/or word omission.

## Author Contributions

AT: Performed the experiments, analyzed the data, wrote the article. C-LG: Contributed rearangements/materials/analysis tools, wrote the article. HP: Contributed rearangements/materials/analysis tools, wrote the article. EB-Q: Contributed rearangements/materials/analysis tools, wrote the article. MPB: Conceived and designed the experiments, performed the experiments, wrote the article.

## Conflict of Interest Statement

The authors declare that the research was conducted in the absence of any commercial or financial relationships that could be construed as a potential conflict of interest.
